# Supplementary material for: Impact of vaccination on the association of COVID-19 with cardiovascular diseases: An OpenSAFELY cohort study
Source: Nat Commun. 2024 Mar 11;15:2173. doi: 10.1038/s41467-024-46497-0 (PMC10928172; doi:10.1038/s41467-024-46497-0)
Supplement: Supplementary file 1 — Supplementary Information [file 41467_2024_46497_MOESM1_ESM.pdf]

Supplementary material

Impact of vaccination on the association of COVID-19 with arterial and venous vascular diseases: an OpenSAFELY cohort study using linked electronic health records

Supplementary methods

Further details of statistical analyses

If there were two events or fewer at any level of a potential confounder, the confounder was excluded from the Cox regression analyses, after levels were aggregated when feasible. To make computations feasible, if the sample size was above four million, the datasets analysed included all individuals with the outcome event or who had COVID-19 during follow up, and a randomly selected subset of those without either the outcome or COVID-19 (“non-exposed controls”). The number of non-exposed controls sampled was based on the number of outcome events: 20 per event for less than 100,000 events, 10 per event for 100,000-500,000 events and 5 per event for >500,000 events. Analyses used inverse probability weights to account for this random sampling, and confidence intervals were derived using robust standard errors. All models were stratified by region, to account for between-region variation.

Absolute excess risks of any ATE and any VTE after COVID-19 diagnosis were derived. The average daily incidence of each outcome before or without COVID-19 over the whole follow-up period was calculated, separately in subgroups defined by age group and sex. The incidence on each day after COVID-19 diagnosis was derived by multiplying the daily incidence by the maximally adjusted HR for that day. Using a life table approach, age- and sex-specific cumulative risks over time, with and without COVID-19 diagnosis, were calculated, subtracting the latter from the former to get absolute excess risks over time after COVID-19 diagnosis versus before or without COVID-19 diagnosis. The overall absolute excess risk was estimated using a weighted sum of the age- and sex-specific excess risks, weighted by the proportions of individuals in age and sex strata in the pre-vaccination cohort.

Individuals with missing age, sex, or deprivation are excluded from the analysis. We included a missing category for ethnicity. All other covariates are defined using the presence versus absence of specific codes, so have no identifiable missing values.

Further details on Information governance and ethical approval

NHS England is the data controller for OpenSAFELY-TPP; TPP is the data processor; all study authors using OpenSAFELY have the approval of NHS England. This implementation of OpenSAFELY is hosted within the TPP environment which is accredited to the ISO 27001 information security standard and is NHS IG Toolkit compliant (https://digital.nhs.uk/data-and-information/looking-after-information/data-security-and-information-governance/data-security-and-protection-toolkit).

Patient data has been pseudonymised for analysis and linkage using industry standard cryptographic hashing techniques; all pseudonymised datasets transmitted for linkage onto OpenSAFELY are encrypted; access to the platform is via a virtual private network (VPN) connection, restricted to a small group of researchers; the researchers hold contracts with NHS England and only access the platform to initiate database queries and statistical models; all database activity is logged; only aggregate statistical outputs leave the platform environment following best practice for anonymisation of results such as statistical disclosure control for low cell counts (https://digital.nhs.uk/data-and-information/information-standards/information-standards-and-data-collections-including-extractions/publications-and-notifications/standards-and-collections/isb1523-anonymisation-standard-for-publishing-health-and-social-care-data).

The OpenSAFELY research platform adheres to the obligations of the UK General Data Protection Regulation (GDPR) and the Data Protection Act 2018. In March 2020, the Secretary of State for Health and Social Care used powers under the UK Health Service (Control of Patient Information) Regulations 2002 (COPI) to require organisations to process confidential patient information for the purposes of protecting public health, providing healthcare services to the public and monitoring and managing the COVID-19 outbreak and incidents of exposure; this sets aside the requirement for patient consent (https://web.archive.org/web/20200421171727/https://www.gov.uk/government/publications/covid-19-notification-to-gps-and-nhs-england-to-share-information). This was extended in November 2022 for the NHS England OpenSAFELY COVID-19 research platform (https://www.gov.uk/government/publications/coronavirus-covid-19-notification-to-organisations-to-share-information/coronavirus-covid-19-notice-under-regulation-34-of-the-health-service-control-of-patient-information-regulations-2002). In some cases of data sharing, the common law duty of confidence is met using, for example, patient consent or support from the Health Research Authority Confidentiality Advisory Group (https://www.hra.nhs.uk/about-us/committees-and-services/confidentiality-advisory-group/).

Taken together, these provide the legal bases to link patient datasets on the OpenSAFELY platform. GP practices, from which the primary care data are obtained, are required to share relevant health information to support the public health response to the pandemic, and have been informed of the OpenSAFELY analytics platform.

Table S1: Distribution of number of days between COVID-19 diagnosis and hospitalisation for 75,667 people in the pre-vaccination cohort who were hospitalised within 28 days of COVID-19 diagnosis.

Number of days	Number of people	Proportion
0	37881	50.06%
1	6278	8.30%
2	3751	4.96%
3	2757	3.64%
4	2677	3.54%
5	2787	3.68%
6	2980	3.94%
7	3030	4.00%
8	2923	3.86%
9	2398	3.17%
10	1922	2.54%
11	1433	1.89%
12	1018	1.35%
13	698	0.92%
14	491	0.65%
15	431	0.57%
16	328	0.43%
17	290	0.38%
18	260	0.34%
19	212	0.28%
20	193	0.26%
21	144	0.19%
22	140	0.19%
23	131	0.17%
24	116	0.15%
25	115	0.15%
26	89	0.12%
27	100	0.13%
28	94	0.12%

Table S2: Patients' medical characteristics in the pre-vaccination, vaccinated and unvaccinated cohorts.

Medical characteristics		Pre-vaccination cohort (Jan 1 2020 to Dec 14 2021)		Vaccinated cohort (June 1 to Dec 14 2021)		Unvaccinated cohort (June 1 to Dec 14 2021)	
		N (%)	COVID-19 diagnoses	N (%)	COVID-19 diagnoses	N (%)	COVID-19 diagnoses
**All**		18,210,937	1,150,299	13,572,399	844,235	3,161,485	162,103
**GP Consultations (in the past year)**	0	4,906,049 (26.9%)	234,801	3,075,265 (22.7%)	179,977	1,690,642 (53.5%)	48,712
	1-6	6,781,543 (37.2%)	449,009	5,323,075 (39.2%)	348,135	883,709 (28.0%)	62,336
	6+	6,523,345 (35.8%)	466,489	5,174,059 (38.1%)	316,123	587,134 (18.6%)	51,055
**Medical history**	All stroke	311,346 (1.7%)	24,611	283,203 (2.1%)	10,458	15,462 (0.5%)	895
	Acute myocardial infarction	456,481 (2.5%)	32,026	415,066 (3.1%)	16,543	21,582 (0.7%)	1,227
	Angina	692,590 (3.8%)	46,663	617,970 (4.6%)	23,980	26,441 (0.8%)	1,542
	Anticoagulation medications	628,605 (3.5%)	43,127	581,792 (4.3%)	23,227	25,599 (0.8%)	1,759
	Antiplatelet medications	2,054,670 (11.3%)	133,027	1,790,224 (13.2%)	75,605	108,586 (3.4%)	7,691
	Cancer	5,386,384 (29.6%)	383,659	4,655,704 (34.3%)	355,899	607,492 (19.2%)	55,519
	Chronic kidney disease	1,141,709 (6.3%)	83,607	1,033,251 (7.6%)	37,977	47,182 (1.5%)	2,936
	Chronic obstructive pulmonary disease	594,413 (3.3%)	39,513	527,658 (3.9%)	20,667	28,676 (0.9%)	1,508
	Combined oral contraceptive pill	2,954,192 (16.2%)	248,502	2,246,588 (16.6%)	217,402	419,921 (13.3%)	39,365
	Dementia	225,378 (1.2%)	37,763	172,881 (1.3%)	6,468	6,114 (0.2%)	370
	Depression	5,172,199 (28.4%)	354,812	4,165,697 (30.7%)	278,033	708,913 (22.4%)	52,714
	Diabetes	1,610,598 (8.8%)	121,394	1,445,448 (10.6%)	77,798	120,772 (3.8%)	9,231
	Healthcare worker	537,203 (2.9%)	73,071	455,039 (3.4%)	36,732	19,241 (0.6%)	2,286
	Heart failure	378,673 (2.1%)	32,434	354,450 (2.6%)	13,831	15,036 (0.5%)	928
	Hormone replacement therapy	1,337,186 (7.3%)	70,349	1,256,529 (9.3%)	64,618	53,901 (1.7%)	3,615
	Hypertension	6,041,285 (33.2%)	382,420	5,275,579 (38.9%)	272,594	461,334 (14.6%)	32,891
	Lipid medications	2,676,353 (14.7%)	157,176	2,323,764 (17.1%)	88,785	97,534 (3.1%)	4,975
	Liver disease	131,402 (0.7%)	8,563	113,791 (0.8%)	5,664	16,124 (0.5%)	828
	Obesity	4,466,692 (24.5%)	330,552	3,758,457 (27.7%)	258,746	477,224 (15.1%)	37,389
	Other arterial embolism	120,783 (0.7%)	8,724	132,299 (1.0%)	5,705	7,790 (0.2%)	467
	Venous thromboembolism	312,998 (1.7%)	22,110	286,893 (2.1%)	14,660	26,636 (0.8%)	2,043

Table S3: Adjusted hazard ratios (95% CI) comparing the incidence of arterial thrombotic, venous thrombotic, and other vascular events after versus before or without a COVID-19 diagnosis, in the pre-vaccination, vaccinated and unvaccinated cohorts, overall and according to COVID-19 severity. Hazard ratios for hospitalised and non-hospitalised COVID-19 are maximally adjusted.

		Time since COVID-19 diagnosis	Pre-vaccination cohort	Vaccinated cohort	Unvaccinated cohort
**Arterial thrombotic events**					
**Acute myocardial infarction**	All, age/sex/region adjusted	Day 0	203.2 (191.1-216.2)	64.9 (58.4-72.2)	192.7 (148.9-249.3)
		1-4 weeks	4.93 (4.56-5.34)	1.98 (1.74-2.24)	9.42 (7.33-12.1)
		5-28 weeks	1.49 (1.40-1.58)	1.06 (0.94-1.20)	2.11 (1.45-3.06)
		29-52 weeks	1.31 (1.23-1.40)	-	-
		53-102 weeks	1.60 (1.45-1.76)	-	-
	Hospitalised COVID-19	Day 0	244.1 (219.0-272.1)	146.0 (117.2-182.0)	220.0 (145.1-333.5)
		1-4 weeks	11.1 (9.95-12.5)	7.73 (6.27-9.54)	18.1 (13.1-25.0)
		5-28 weeks	1.64 (1.44-1.87)	1.25 (0.85-1.84)	1.54 (0.68-3.45)
		29-52 weeks	1.30 (1.12-1.52)	-	-
		53-102 weeks	1.43 (1.18-1.72)	-	-
	Non-hospitalised COVID-19	Day 0	151.0 (140.2-162.6)	54.0 (47.8-61.0)	116.5 (83.7-162.2)
		1-4 weeks	2.67 (2.40-2.98)	1.39 (1.19-1.62)	3.68 (2.48-5.44)
		5-28 weeks	1.24 (1.17-1.33)	1.02 (0.90-1.16)	1.62 (1.07-2.45)
		29-52 weeks	1.12 (1.05-1.21)	-	-
		53-102 weeks	1.25 (1.12-1.40)	-	-
**Ischaemic stroke**	All, age/sex/region adjusted	Day 0	262.6 (248.8-277.1)	84.1 (76.6-92.4)	290.1 (232.1-362.6)
		1-4 weeks	4.70 (4.33-5.10)	2.22 (1.97-2.50)	9.76 (7.53-12.6)
		5-28 weeks	1.63 (1.54-1.73)	1.14 (1.01-1.29)	1.66 (1.06-2.59)
		29-52 weeks	1.26 (1.18-1.35)	-	-
		53-102 weeks	1.48 (1.35-1.63)	-	-
	Hospitalised COVID-19	Day 0	177.5 (156.6-201.1)	88.6 (67.4-116.5)	299.3 (207.4-431.8)
		1-4 weeks	9.92 (8.80-11.2)	6.40 (5.12-8.01)	15.0 (10.5-21.4)
		5-28 weeks	1.92 (1.69-2.17)	2.11 (1.57-2.84)	1.60 (0.71-3.60)
		29-52 weeks	1.31 (1.12-1.53)	-	-
		53-102 weeks	1.25 (1.03-1.52)	-	-
	Non-hospitalised COVID-19	Day 0	227.2 (213.8-241.4)	76.1 (68.7-84.3)	178.2 (133.5-237.9)
		1-4 weeks	2.57 (2.30-2.88)	1.65 (1.43-1.91)	4.60 (3.15-6.73)
		5-28 weeks	1.31 (1.23-1.40)	0.99 (0.87-1.12)	1.18 (0.70-2.00)
		29-52 weeks	1.05 (0.97-1.13)	-	-
		53-102 weeks	1.13 (1.02-1.27)	-	-
**Venous thrombotic events**					
**Pulmonary embolism**	All, age/sex/region adjusted	Day 0	655.5 (622.7-690.1)	146.7 (132.2-162.9)	1467 (1264-1702)
		1-4 weeks	35.1 (33.6-36.6)	9.87 (9.09-10.7)	106.0 (93.2-120.5)
		5-28 weeks	2.20 (2.06-2.36)	1.87 (1.64-2.14)	5.51 (3.99-7.62)
		29-52 weeks	1.13 (1.03-1.24)	-	-
		53-102 weeks	1.32 (1.15-1.53)	-	-
	Hospitalised COVID-19	Day 0	1910 (1777-2053)	829.0 (710.4-967.5)	6845 (5706-82115)
		1-4 weeks	177.5 (168.2-187.4)	93.5 (83.9-104.2)	535.5 (455.0-630.2)
		5-28 weeks	5.73 (5.11-6.43)	8.31 (6.49-10.6)	14.9 (9.22-24.1)
		29-52 weeks	1.58 (1.27-1.96)	-	-
		53-102 weeks	1.49 (1.12-1.99)	-	-
	Non-hospitalised COVID-19	Day 0	355.2 (330.7-381.5)	78.3 (68.0-90.3)	279.9 (212.8-368.0)
		1-4 weeks	9.34 (8.60-10.1)	2.84 (2.44-3.30)	17.9 (14.1-22.5)
		5-28 weeks	1.53 (1.41-1.65)	1.31 (1.12-1.53)	2.47 (1.61-3.78)
		29-52 weeks	0.96 (0.87-1.06)	-	-
		53-102 weeks	1.05 (0.89-1.23)	-	-
**Deep vein thrombosis**	All, age/sex/region adjusted	Day 0	140.1 (127.9-153.3)	28.6 (23.6-34.7)	93.8 (68.8-127.8)
		1-4 weeks	7.43 (6.86-8.03)	2.48 (2.17-2.85)	8.23 (6.55-10.4)
		5-28 weeks	1.94 (1.83-2.07)	1.56 (1.38-1.77)	2.28 (1.63-3.18)
		29-52 weeks	1.28 (1.18-1.38)	-	-
		53-102 weeks	1.53 (1.35-1.74)	-	-

		Time since COVID-19 diagnosis	Pre-vaccination cohort	Vaccinated cohort	Unvaccinated cohort
	Hospitalised COVID-19	Day 0	310.0 (267.0-360.0)	156.3 (113.3-215.8)	263.9 (53.1-1310.6)
		1-4 weeks	22.6 (20.0-25.6)	10.5 (8.00-13.8)	38.2 (17.6-82.7)
		5-28 weeks	3.61 (3.16-4.12)	3.55 (2.50-5.05)	2.33 (0.32-17.2)
		29-52 weeks	1.34 (1.07-1.67)	-	-
		53-102 weeks	1.29 (0.96-1.74)	-	-
	Non-hospitalised COVID-19	Day 0	91.5 (81.7-102.4)	18.5 (14.6-23.5)	46.0 (30.3-69.8)
		1-4 weeks	4.29 (3.86-4.76)	1.85 (1.58-2.17)	3.59 (2.60-4.94)
		5-28 weeks	1.54 (1.44-1.65)	1.34 (1.18-1.54)	1.07 (0.70-1.63)
		29-52 weeks	1.14 (1.05-1.24)	-	-
		53-102 weeks	1.27 (1.10-1.46)	-	-
**Other cardiovascular events**					
**Heart failure**	All, age/sex/region adjusted	Day 0	309.6 (300.8-318.5)	81.8 (78.0-85.8)	262.8 (229.3-301.2)
		1-4 weeks	5.24 (5.00-5.48)	2.59 (2.45-2.74)	9.50 (8.10-11.2)
		5-28 weeks	1.61 (1.55-1.67)	1.16 (1.09-1.23)	2.27 (1.78-2.88)
		29-52 weeks	1.25 (1.20-1.30)	-	-
		53-102 weeks	1.26 (1.19-1.34)	-	-
	Hospitalised COVID-19	Day 0	448.9 (430.5-468.2)	181.5 (168.9-195.1)	334.5 (279.4-400.3)
		1-4 weeks	17.3 (16.2-18.4)	14.8 (13.6-16.1)	23.1 (18.7-28.6)
		5-28 weeks	2.28 (2.11-2.47)	2.02 (1.71-2.39)	2.75 (1.73-4.36)
		29-52 weeks	1.29 (1.16-1.44)	-	-
		53-102 weeks	1.12 (0.98-1.29)	-	-
	Non-hospitalised COVID-19	Day 0	170.7 (164.2-177.5)	48.8 (45.8-52.1)	90.5 (71.3-114.8)
		1-4 weeks	1.90 (1.75-2.05)	1.17 (1.07-1.27)	2.13 (1.51-2.99)
		5-28 weeks	1.28 (1.23-1.33)	1.05 (0.98-1.12)	1.57 (1.17-2.10)
		29-52 weeks	1.06 (1.01-1.11)	-	-
		53-102 weeks	1.02 (0.95-1.10)	-	-
**Angina**	All, age/sex/region adjusted	Day 0	166.5 (159.3-174.0)	48.8 (45.3-52.4)	220.9 (183.1-266.5)
		1-4 weeks	4.79 (4.54-5.06)	2.19 (2.03-2.35)	8.61 (7.01-10.6)
		5-28 weeks	1.39 (1.34-1.45)	1.20 (1.12-1.28)	1.81 (1.31-2.50)
		29-52 weeks	1.27 (1.21-1.33)	-	-
		53-102 weeks	1.41 (1.31-1.52)	-	-
	Hospitalised COVID-19	Day 0	314.3 (296.0-333.7)	182.5 (165.3-201.6)	296.1 (236.4-370.9)
		1-4 weeks	15.7 (14.6-16.8)	13.5 (12.2-15.0)	19.5 (15.3-24.8)
		5-28 weeks	1.24 (1.11-1.38)	1.25 (0.97-1.60)	1.33 (0.66-2.71)
		29-52 weeks	1.05 (0.93-1.20)	-	-
		53-102 weeks	1.17 (1.00-1.37)	-	-
	Non-hospitalised COVID-19	Day 0	85.5 (80.3-91.0)	29.0 (26.4-31.7)	68.2 (49.4-94.3)
		1-4 weeks	1.75 (1.60-1.92)	0.99 (0.89-1.10)	1.81 (1.18-2.79)
		5-28 weeks	1.22 (1.17-1.28)	1.10 (1.03-1.19)	1.33 (0.92-1.92)
		29-52 weeks	1.12 (1.06-1.17)	-	-
		53-102 weeks	1.16 (1.07-1.26)	-	-
**Transient ischaemic attack**	All, age/sex/region adjusted	Day 0	51.2 (43.0-60.9)	22.4 (17.3-29.0)	†
		1-4 weeks	2.20 (1.85-2.62)	1.31 (1.05-1.64)	†
		5-28 weeks	1.34 (1.22-1.46)	1.21 (1.02-1.43)	†
		29-52 weeks	1.22 (1.10-1.34)	-	-
		53-102 weeks	1.26 (1.08-1.46)	-	-
	Hospitalised COVID-19	Day 0	63.9 (45.9-89.1)	†	†
		1-4 weeks	2.87 (2.03-4.06)	†	†
		5-28 weeks	1.34 (1.07-1.68)	†	†
		29-52 weeks	1.38 (1.09-1.74)	-	-
		53-102 weeks	0.95 (0.67-1.35)	-	-
	Non-hospitalised COVID-19	Day 0	41.6 (33.9-51.1)	18.2 (13.5-24.6)	†
		1-4 weeks	1.83 (1.50-2.24)	1.08 (0.84-1.39)	†
		5-28 weeks	1.21 (1.10-1.34)	1.07 (0.89-1.29)	†
		29-52 weeks	1.08 (0.97-1.20)	-	-
		53-102 weeks	1.15 (0.97-1.36)	-	-

		Time since COVID-19 diagnosis	Pre-vaccination cohort	Vaccinated cohort	Unvaccinated cohort
**Subarachnoid haemorrhage and haemorrhagic stroke**	All, age/sex/region adjusted	Day 0	328.8 (293.7-367.9)	97.3 (78.8-120.1)	†
		1-4 weeks	5.12 (4.31-6.09)	2.39 (1.81-3.14)	†
		5-28 weeks	1.59 (1.40-1.81)	1.23 (0.93-1.61)	†
		29-52 weeks	1.45 (1.26-1.67)	-	-
		53-102 weeks	1.69 (1.37-2.10)	-	-
	Hospitalised COVID-19	Day 0	215.0 (161.4-286.3)	†	†
		1-4 weeks	16.3 (12.9-20.4)	†	†
		5-28 weeks	2.04 (1.53-2.73)	†	†
		29-52 weeks	1.62 (1.15-2.30)	-	-
		53-102 weeks	1.57 (1.01-2.43)	-	-
	Non-hospitalised COVID-19	Day 0	302.3 (267.4-341.9)	93.0 (74.5-116.1)	†
		1-4 weeks	2.28 (1.74-2.97)	1.91 (1.40-2.61)	†
		5-28 weeks	1.35 (1.17-1.55)	1.12 (0.84-1.49)	†
		29-52 weeks	1.27 (1.09-1.48)	-	-
		53-102 weeks	1.37 (1.08-1.75)	-	-

† Insufficient events for estimation

Table S4: Ratios of adjusted hazard ratios (95% CI) comparing associations of COVID-19 diagnosis with arterial thrombotic and venous thrombotic events between the vaccinated, unvaccinated and pre-vaccination cohorts.

Outcome	Cohort comparison	Weeks since COVID-19	HR ratio (95% CI)
Arterial thrombotic events	Vaccinated / Unvaccinated	1-4	0.28 (0.25-0.32)
		5-28	0.70 (0.52-0.94)
	Vaccinated / Pre-vaccination	1-4	0.36 (0.33-0.38)
		5-28	0.73 (0.66-0.82)
	Unvaccinated / Pre-vaccination	1-4	1.25 (1.11-1.42)
		5-28	1.05 (0.79-1.39)
Venous thrombotic events	Vaccinated / Unvaccinated	1-4	0.17 (0.15-0.19)
		5-28	0.63 (0.49-0.80)
	Vaccinated / Pre-vaccination	1-4	0.24 (0.23-0.26)
		5-28	0.61 (0.55-0.68)
	Unvaccinated / Pre-vaccination	1-4	1.44 (1.31-1.57)
		5-28	0.97 (0.76-1.23)

Table S5: Adjusted hazard ratios (95% CI) comparing the incidence of arterial thrombotic and venous thrombotic events after versus before or without a COVID-19 diagnosis, in the unvaccinated cohort, overall and according to COVID-19 severity. Comparing results from the main analysis and to results where censoring at vaccination was removed. Estimated hazard ratios for weeks 1-4 include the day of COVID-19 diagnosis (day 0).

	Weeks since COVID-19	Unvaccinated cohort	Unvaccinated cohort without censoring at vaccination
**All arterial thrombotic events**			
All, age/sex/region adjusted	1-4	21.4 (19.0-24.1)	19.3 (17.2-21.6)
	5-28	1.94 (1.47-2.57)	1.74 (1.34-2.26)
All, maximally adjusted	1-4	17.2 (15.3-19.4)	15.9 (14.2-17.8)
	5-28	1.55 (1.17-2.05)	1.44 (1.11-1.87)
Hospitalised COVID-19	1-4	32.8 (27.5-39.2)	31.0 (26.1-36.9)
	5-28	1.56 (0.88-2.78)	1.58 (0.94-2.64)
Non-hospitalised COVID-19	1-4	10.9 (9.27-12.9)	10.1 (8.65-11.8)
	5-28	1.45 (1.06-2.00)	1.30 (0.97-1.76)
**All venous thrombotic events**			
All, age/sex/region adjusted	1-4	56.8 (52.3-61.8)	50.0 (46.1-54.2)
	5-28	3.21 (2.55-4.05)	2.84 (2.28-3.53)
All, maximally adjusted	1-4	45.4 (41.7-49.4)	40.7 (37.5-44.2)
	5-28	2.44 (1.94-3.07)	2.17 (1.74-2.69)
Hospitalised COVID-19	1-4	303 (269-341)	286 (255-321)
	5-28	7.58 (5.12-11.2)	6.43 (4.38-9.44)
Non-hospitalised COVID-19	1-4	12.0 (10.3-13.9)	11.0 (9.51-12.6)
	5-28	1.63 (1.22-2.17)	1.54 (1.19-2.01)

Table S6: Adjusted hazard ratios (95% CI) comparing the incidence of arterial thrombotic and venous thrombotic events after versus before or without a COVID-19 diagnosis during the first 28 days after COVID-19 diagnosis, in the pre-vaccination and vaccinated cohorts, overall and according to COVID-19 severity.

		Adjusted hazard ratio (95% CI)		
	**Days since COVID-19**	**Pre-vaccination cohort**	**Vaccinated cohort**	**Unvaccinated cohort**
**Arterial thrombotic events**				
All	0	200.2 (192.3-208.4)	71.8 (66.9-76.9)	248.7 (212.0-291.6)
	1-6	6.89 (6.31-7.53)	3.09 (2.69-3.56)	19.9 (15.7-25.2)
	7-13	5.20 (4.72-5.72)	2.28 (1.95-2.67)	11.0 (8.12-14.9)
	14-20	3.45 (3.06-3.90)	1.73 (1.44-2.08)	7.28 (4.94-10.7)
	21-27	2.11 (1.81-2.47)	1.25 (1.00-1.56)	2.13 (1.01-4.48)
Hospitalised COVID-19	0	228.2 (210.9-247.0)	191.3 (162.8-224.8)	481.6 (378.1-613.6)
	1-6	19.4 (17.3-21.8)	22.5 (18.5-27.5)	57.1 (42.3-77.1)
	7-13	14.5 (12.7-16.6)	10.5 (7.89-14.0)	37.5 (25.9-54.2)
	14-20	7.97 (6.57-9.66)	5.87 (3.90-8.83)	18.0 (10.2-31.7)
	21-27	4.33 (3.30-5.68)	4.80 (2.98-7.73)	6.76 (2.53-18.1)
Non-hospitalised COVID-19	0	193.5 (184.9-202.6)	63.7 (58.9-68.9)	186.0 (151.3-228.6)
	1-6	3.57 (3.11-4.10)	1.73 (1.42-2.11)	10.0 (6.94-14.5)
	7-13	3.07 (2.67-3.54)	1.77 (1.47-2.13)	4.46 (2.63-7.55)
	14-20	2.54 (2.17-2.96)	1.51 (1.23-1.85)	4.80 (2.84-8.13)
	21-27	1.70 (1.40-2.05)	1.06 (0.83-1.37)	1.11 (0.36-3.44)
**Venous thrombotic events**				
All	0	313.3 (299.7-327.4)	72.3 (66.2-79.0)	459.5 (405.6-520.5)
	1-6	23.0 (21.5-24.6)	5.50 (4.81-6.28)	54.1 (46.6-62.8)
	7-13	21.4 (20.1-22.8)	6.85 (6.11-7.67)	47.7 (41.0-55.5)
	14-20	15.1 (14.0-16.2)	4.48 (3.87-5.17)	27.2 (22.2-33.4)
	21-27	7.40 (6.65-8.23)	2.39 (1.95-2.93)	11.1 (7.99-15.3)
Hospitalised COVID-19	0	986.9 (924.9-1053.0)	669.2 (584.8-765.8)	3151.6 (2734.2-3632.7)
	1-6	123.0 (113.9-132.9)	95.3 (81.8-111.0)	450.6 (383.8-529.0)
	7-13	124.1 (115.0-133.9)	103.3 (89.5-119.3)	391.3 (330.1-463.7)
	14-20	67.6 (60.9-75.1)	43.2 (34.2-54.7)	148.3 (112.1-196.2)
	21-27	30.0 (25.6-35.1)	25.9 (18.9-35.7)	47.7 (28.6-79.3)
Non-hospitalised COVID-19	0	196.1 (184.9-208.1)	45.5 (40.6-51.0)	126.1 (100.4-158.3)
	1-6	5.74 (4.99-6.60)	1.34 (1.01-1.76)	6.91 (4.61-10.4)
	7-13	5.84 (5.13-6.64)	2.66 (2.21-3.21)	8.77 (6.21-12.4)
	14-20	7.86 (7.04-8.78)	2.93 (2.44-3.52)	13.7 (10.2-18.3)
	21-27	4.50 (3.89-5.21)	1.49 (1.14-1.94)	6.95 (4.56-10.6)

Table S7: Maximally adjusted hazard ratios (95% CI) comparing the incidence of arterial thrombotic events after versus before or without a COVID-19 diagnosis, in the pre-vaccination, vaccinated and unvaccinated cohorts, by subgroup. Estimated hazard ratios for weeks 1-4 include the day of COVID-19 diagnosis (day 0).

Subgroup		Weeks since COVID-19	Pre-vaccination cohort	Vaccinated cohort	Unvaccinated cohort
Age group	18-39	1-4	6.37 (4.99-8.12)	†	†
		5-28	1.21 (0.98-1.50)	†	†
		29-52	1.07 (0.85-1.34)	-	-
		53-102	1.03 (0.64-1.64)	-	-
	40-59	1-4	8.62 (7.98-9.30)	2.35 (2.05-2.71)	12.8 (10.5-15.6)
		5-28	1.19 (1.10-1.29)	0.83 (0.70-0.98)	1.12 (0.72-1.76)
		29-52	1.12 (1.03-1.22)	-	-
		53-102	1.30 (1.12-1.51)	-	-
	60-79	1-4	15.9 (15.1-16.6)	4.84 (4.47-5.24)	25.3 (21.1-30.4)
		5-28	1.42 (1.34-1.51)	1.11 (0.99-1.25)	1.44 (0.85-2.45)
		29-52	1.12 (1.04-1.20)	-	-
		53-102	1.33 (1.19-1.47)	-	-
	80-110	1-4	13.0 (12.2-13.8)	8.73 (7.99-9.54)	†
		5-28	1.42 (1.32-1.54)	1.19 (1.00-1.41)	†
		29-52	1.07 (0.97-1.18)	-	-
		53-102	1.06 (0.94-1.19)	-	-
Sex	Female	1-4	11.2 (10.6-11.9)	4.94 (4.53-5.39)	13.4 (10.9-16.3)
		5-28	1.08 (1.00-1.16)	1.02 (0.89-1.16)	2.21 (1.55-3.16)
		29-52	1.34 (1.26-1.43)	-	-
		53-102	1.09 (0.98-1.21)	-	-
	Male	1-4	13.5 (12.9-14.1)	4.78 (4.45-5.13)	18.6 (16.0-21.6)
		5-28	1.36 (1.29-1.43)	1.10 (0.99-1.23)	0.95 (0.60-1.49)
		29-52	1.14 (1.08-1.21)	-	-
		53-102	1.31 (1.20-1.44)	-	-
Ethnicity	White	1-4	12.5 (12.1-13.0)	4.89 (4.61-5.18)	15.0 (13.1-17.2)
		5-28	1.37 (1.31-1.43)	1.09 (1.00-1.19)	1.49 (1.09-2.03)
		29-52	1.14 (1.09-1.20)	-	-
		53-102	1.21 (1.13-1.31)	-	-
	Black	1-4	15.5 (12.4-19.3)	†	†
		5-28	1.13 (0.83-1.54)	†	†
		29-52	0.91 (0.64-1.30)	-	-
		53-102	1.19 (0.75-1.88)	-	-
	South Asian	1-4	12.9 (11.6-14.4)	4.52 (3.56-5.73)	-
		5-28	1.23 (1.08-1.40)	0.83 (0.57-1.22)	-
		29-52	1.00 (0.87-1.16)	-	-
		53-102	1.19 (0.95-1.48)	-	-
	Other ethnic groups	1-4	12.9 (9.58-17.2)	†	†
		5-28	1.44 (1.01-2.07)	†	†
		29-52	0.90 (0.57-1.42)	-	-
		53-102	1.02 (0.52-1.96)	-	-
	Mixed	1-4	16.9 (11.9-23.9)	†	†
		5-28	1.96 (1.29-2.99)	†	†
		29-52	0.80 (0.41-1.55)	-	-
		53-102	2.02 (1.00-4.09)	-	-
Prior history of event	Yes	1-4	10.5 (9.88-11.2)	4.73 (4.28-5.22)	8.58 (6.48-11.4)
		5-28	1.30 (1.20-1.40)	1.06 (0.91-1.23)	0.65 (0.31-1.37)
		29-52	1.13 (1.03-1.23)	-	-
		53-102	1.24 (1.11-1.39)	-	-
	No	1-4	13.7 (13.1-14.2)	5.08 (4.76-5.43)	21.2 (18.5-24.2)
		5-28	1.39 (1.32-1.46)	1.09 (0.99-1.21)	1.91 (1.41-2.59)
		29-52	1.13 (1.07-1.19)	-	-
		53-102	1.23 (1.13-1.33)	-	-

† insufficient events for estimation

Table S8: Maximally adjusted hazard ratios (95% CI) comparing the incidence of venous thrombotic events after versus before or without a COVID-19 diagnosis, in the pre-vaccination, vaccinated and unvaccinated cohorts, by subgroup. Estimated hazard ratios for weeks 1-4 include the day of COVID-19 diagnosis (day 0).

Subgroup		Weeks since COVID-19	Pre-vaccination cohort	Vaccinated cohort	Unvaccinated cohort
Age group	18-39	1-4	15.2 (13.7-16.9)	2.83 (2.14-3.75)	18.7 (15.7-22.3)
		5-28	1.44 (1.28-1.63)	1.50 (1.13-1.99)	1.41 (0.90-2.21)
		29-52	1.08 (0.94-1.25)	-	-
		53-102	1.29 (0.98-1.70)	-	-
	40-59	1-4	29.2 (27.7-30.8)	5.11 (4.57-5.71)	54.7 (48.2-62.1)
		5-28	1.65 (1.53-1.79)	1.19 (1.00-1.40)	2.52 (1.80-3.54)
		29-52	1.01 (0.91-1.11)	-	-
		53-102	1.14 (0.94-1.36)	-	-
	60-79	1-4	39.6 (37.9-41.5)	10.0 (9.24-10.8)	94.8 (79.6-113.0)
		5-28	2.35 (2.19-2.52)	1.75 (1.53-2.01)	3.78 (2.30-6.20)
		29-52	1.17 (1.06-1.29)	-	-
		53-102	1.31 (1.13-1.53)	-	-
	80-110	1-4	22.5 (20.7-24.4)	14.1 (12.5-16.0)	†
		5-28	2.07 (1.85-2.31)	1.94 (1.54-2.45)	†
		29-52	1.18 (1.01-1.38)	-	-
		53-102	1.09 (0.89-1.32)	-	-
Sex	Female	1-4	20.7 (19.8-21.7)	6.01 (5.49-6.57)	25.5 (22.2-29.2)
		5-28	1.75 (1.64-1.86)	1.45 (1.28-1.65)	2.15 (1.56-2.97)
		29-52	1.12 (1.03-1.21)	-	-
		53-102	1.15 (1.01-1.31)	-	-
	Male	1-4	36.1 (34.7-37.6)	9.41 (8.74-10.1)	69.7 (62.3-78.1)
		5-28	1.99 (1.87-2.12)	1.59 (1.40-1.80)	2.55 (1.81-3.58)
		29-52	1.06 (0.97-1.15)	-	-
		53-102	1.24 (1.07-1.42)	-	-
Ethnicity	White	1-4	25.7 (24.8-26.5)	7.65 (7.21-8.11)	37.5 (34.0-41.3)
		5-28	1.86 (1.77-1.95)	1.55 (1.41-1.70)	2.27 (1.76-2.93)
		29-52	1.09 (1.03-1.16)	-	-
		53-102	1.18 (1.07-1.31)	-	-
	Black	1-4	48.7 (41.7-56.8)	†	76.9 (34.4-172.1)
		5-28	1.88 (1.42-2.50)	†	3.32 (0.39-28.6)
		29-52	0.80 (0.53-1.21)	-	-
		53-102	1.80 (1.14-2.85)	-	-
	South Asian	1-4	46.0 (41.1-51.4)	†	81.7 (58.0-115.1)
		5-28	1.90 (1.58-2.28)	†	†
		29-52	1.13 (0.90-1.43)	-	-
		53-102	1.43 (0.99-2.07)	-	-
	Other ethnic groups	1-4	66.7 (52.6-84.7)	†	†
		5-28	1.34 (0.8-2.26)	†	†
		29-52	2.98 (2.06-4.31)	-	-
		53-102	0.52 (0.13-2.11)	-	-
	Mixed	1-4	36.3 (27.0-48.8)	†	†
		5-28	1.25 (0.73-2.13)	†	†
		29-52	1.22 (0.71-2.09)	-	-
		53-102	†	-	-
Prior history of event	Yes	1-4	12.6 (11.4-13.8)	4.06 (3.48-4.74)	6.86 (5.12-9.20)
		5-28	1.47 (1.31-1.65)	1.27 (1.04-1.55)	1.24 (0.77-1.99)
		29-52	1.01 (0.87-1.18)	-	-
		53-102	1.20 (0.96-1.50)	-	-
	No	1-4	32.3 (31.3-33.3)	8.94 (8.40-9.50)	65.8 (60.0-72.2)
		5-28	1.97 (1.88-2.07)	1.61 (1.46-1.78)	2.83 (2.17-3.69)
		29-52	1.11 (1.05-1.19)	-	-
		53-102	1.20 (1.08-1.34)	-	-

† insufficient events for estimation

Table S9: Estimated excess events at 28 weeks for pre-vaccination, vaccinated and unvaccinated cohorts

Outcome		Pre-vaccination cohort	Vaccinated cohort	Unvaccinated cohort
Arterial thrombotic events	Total excess events	4,546	1,491	374
	Total post exposure follow-up (years)	522,421	158,306	25,578
	Excess events per 100,000 COVID-19 diagnosis	642	229	718
Venous thrombotic events	Total excess events	6,360	1,991	1,083
	Total post exposure follow-up (years)	522,573	158,379	25,472
	Excess events per 100,000 COVID-19 diagnosis	797	270	1094

Table S10: List of Covariates

The following potential confounders were defined using the most recent data prior to the study start date:

Confounder	Type	Definition	Data sources
Sex*	Categorical	Male, Female	Primary care (see: https://docs.opensafely.org/study-def-variables/#cohortextractor.patients.sex)
Age	Continuous	Modelled as age in years using a restricted cubic spline with 3 knots at the 10th, 50th and 90th percentiles	All
Ethnicity	Categorical	1: White<br>2: Mixed<br>3: South Asian<br>4: Black<br>5: Other<br>6: Missing/Unknown	All
Deprivation	Categorical	Index of Multiple Deprivation 2019 quintiles	Index of Multiple Deprivation 2019
Region	Categorical	East<br>East Midlands<br>London<br>North East<br>North West<br>South East<br>South West<br>West Midlands<br>Yorkshire/Humber	Primary care
Patient-GP contact	Continuous	Number of primary care contacts in the year prior to index date	Primary care
Smoking status	Categorial	N: Never smoker<br>E:-Ex-smoker<br>S: Current smoker<br>M: Missing/Unknown	Primary care
Obesity	Binary	1 if BMI>=30 or coded diagnosis for obesity; 0 otherwise	Primary care, HES APC
Acute myocardial infarction	Binary	1 if diagnosis present; 0 otherwise	Primary care, HES APC
All stroke	Binary	1 if diagnosis present; 0 otherwise	Primary care, HES APC
Other arterial embolism	Binary	1 if diagnosis present; 0 otherwise	Primary care, HES APC
Venous thromboembolism events	Binary	1 if diagnosis present; 0 otherwise	Primary care, HES APC
Heart failure	Binary	1 if diagnosis present; 0 otherwise	Primary care, HES APC
Angina	Binary	1 if diagnosis present; 0 otherwise	Primary care, HES APC

Dementia	Binary	1 if diagnosis present; 0 otherwise	Primary care, HES APC
Liver disease	Binary	1 if diagnosis present; 0 otherwise	Primary care, HES APC
Chronic kidney disease	Binary	1 if diagnosis present; 0 otherwise	Primary care, HES APC
Cancer	Binary	1 if diagnosis present; 0 otherwise	Primary care, HES APC
Hypertension	Binary	1 if diagnosis or prescription present; 0 otherwise	Primary care, HES APC
Diabetes	Binary	1 if diagnosis or prescription present; 0 otherwise	Primary care, HES APC
Depression	Binary	1 if diagnosis present; 0 otherwise	Primary care, HES APC
Chronic obstructive pulmonary disease	Binary	1 if diagnosis present; 0 otherwise	Primary care, HES APC
Lipid lowering medications	Binary	1 if prescription present; 0 otherwise	Primary care
Antiplatelet medications	Binary	1 if prescription present; 0 otherwise	Primary care
Anticoagulation medications	Binary	1 if prescription present; 0 otherwise	Primary care
Combined oral contraceptive pill	Binary	1 if prescription present; 0 otherwise	Primary care
Hormone replacement therapy	Binary	1 if prescription present; 0 otherwise	Primary care
Healthcare worker*	Binary	1 if healthcare worker; 0 otherwise	NHS England COVID-19 data store (see: https://docs.opensafely.org/study-def-variables/#cohortextractor.patients.with_healthcare_worker_flag_on_covid_vaccine_record)
Care home resident	Binary	1 if care home resident; 0 otherwise	Address matching CQC database (see: https://docs.opensafely.org/study-def-variables/#cohortextractor.patients.care_home_status_as_of)

* These variables are derived once per patient without a date specification so are an exception to ‘most recent data prior to the study start date’

Figure S1: Maximally adjusted hazard ratios and 95% CIs comparing the incidence of arterial thrombotic events after versus before or without a COVID-19 diagnosis, in the pre-vaccination, vaccinated and unvaccinated cohorts, overall and by COVID-19 severity. Sensitivity analysis comparing results for events identified through primary diagnosis only with those identified using codes in any position.

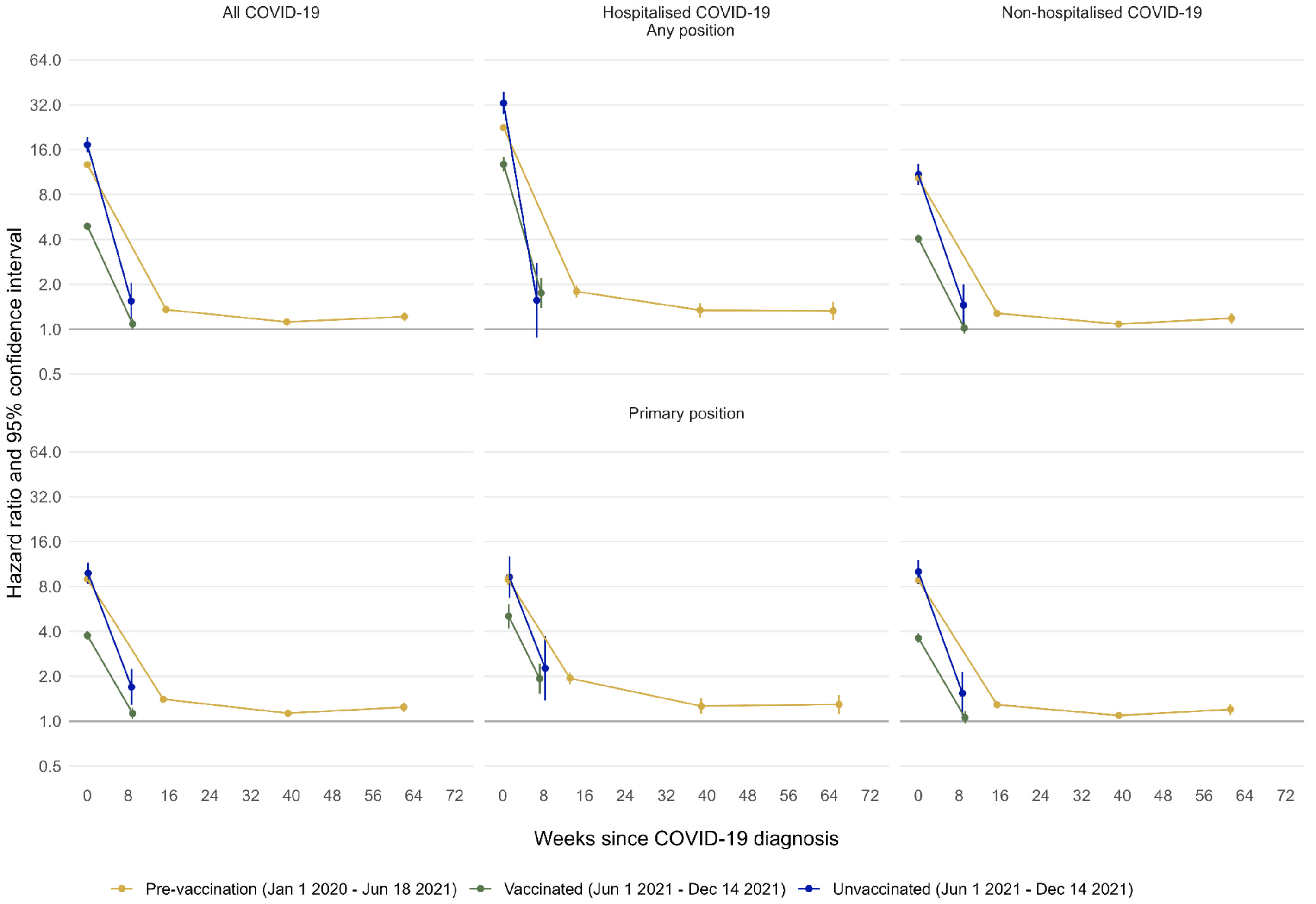

Figure S1 legend: Upper panels: estimated hazard ratios for arterial thrombotic events in any position. Lower panels: estimated hazard ratios for arterial thrombotic events in primary position. Left panels: all COVID-19 diagnoses: Middle panels: hospitalised COVID-19. Right panels: non-hospitalised COVID-19.

The numbers of people in the pre-vaccination, vaccinated and unvaccinated cohorts were 18,210,937; 13,572,399 and 3,161,485 respectively. The numbers of COVID-19 diagnoses were 1,150,299 (75,667 hospitalised) in the pre-vaccination cohort, 844,235 (15,342 hospitalised) in the vaccinated cohort and 162,103 (9,250 hospitalised) in the unvaccinated cohort. Estimated hazard ratios are plotted at the median time of the outcome event within each follow up period in each cohort. Vertical lines around estimated hazard ratios are 95% confidence intervals, derived using Cox regression. Estimated hazard ratios for weeks 1-4 include the day of COVID-19 diagnosis (day 0).

Figure S2: Maximally adjusted hazard ratios and 95% CIs comparing the incidence of venous thrombotic events after versus before or without a COVID-19 diagnosis, in the pre-vaccination, vaccinated and unvaccinated cohorts, overall and by COVID-19 severity. Sensitivity analysis comparing results for events identified through primary diagnosis only with those identified using codes in any position.

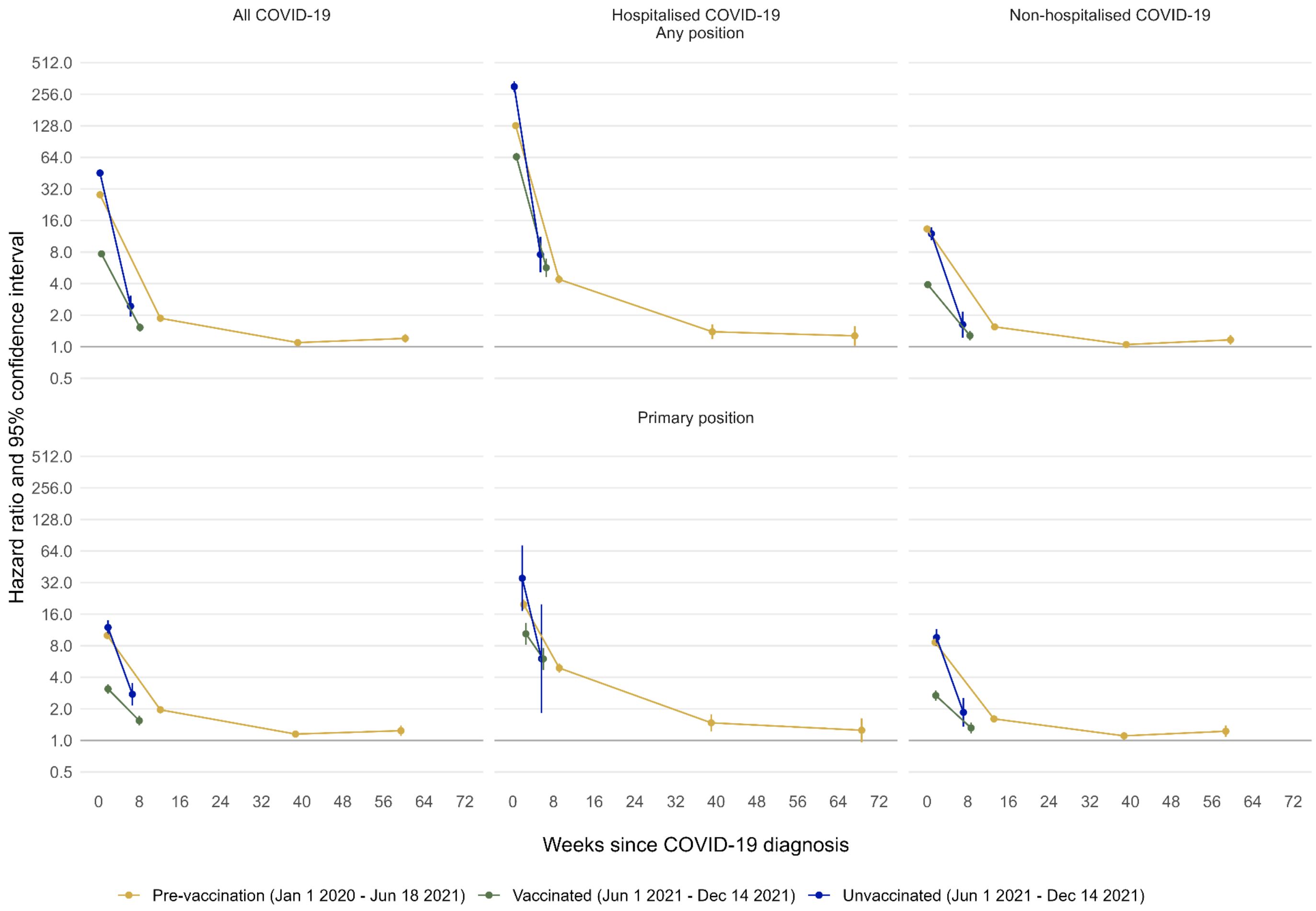

Figure S2 legend: Upper panels: estimated hazard ratios for venous thrombotic events in any position. Lower panels: estimated hazard ratios for venous thrombotic events in primary position. Left panels: all COVID-19 diagnoses: Middle panels: hospitalised COVID-19. Right panels: non-hospitalised COVID-19.

The numbers of people in the pre-vaccination, vaccinated and unvaccinated cohorts were 18,210,937; 13,572,399 and 3,161,485 respectively. The numbers of COVID-19 diagnoses were 1,150,299 (75,667 hospitalised) in the pre-vaccination cohort, 844,235 (15,342 hospitalised) in the vaccinated cohort and 162,103 (9,250 hospitalised) in the unvaccinated cohort. Estimated hazard ratios are plotted at the median time of the outcome event within each follow up period in each cohort. Vertical lines around estimated hazard ratios are 95% confidence intervals, derived using Cox regression. Estimated hazard ratios for weeks 1-4 include the day of COVID-19 diagnosis (day 0).

Figure S3: Maximally adjusted hazard ratios and 95% CIs comparing the incidence of arterial thrombotic events after versus before or without a COVID-19 diagnosis, in the pre-vaccination, vaccinated and unvaccinated cohorts, by subgroup.

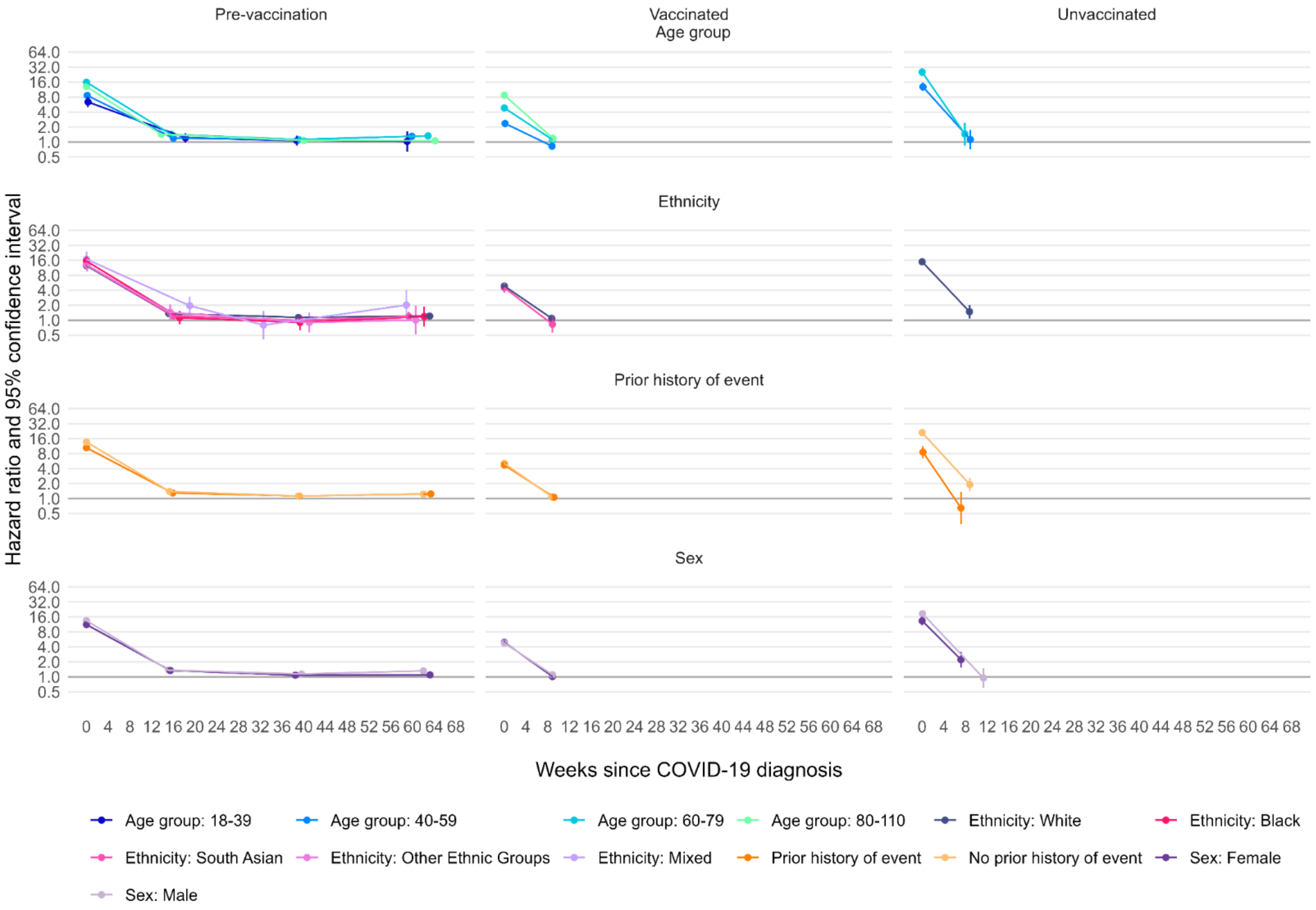

Figure S3 legend: Upper panels: Age group. Second row panels: Ethnicity. Third row panels: prior history of events. Lower panels: Sex. Left panels: pre-vaccination: Middle panels: vaccinated. Right panels: unvaccinated.

The numbers of people in the pre-vaccination, vaccinated and unvaccinated cohorts were 18,210,937; 13,572,399 and 3,161,485 respectively. The numbers of COVID-19 diagnoses were 1,150,299 in the pre-vaccination cohort, 844,235 in the vaccinated cohort and 162,103 in the unvaccinated cohort. Estimated hazard ratios are plotted at the median time of the outcome event within each follow up period in each cohort. Vertical lines around estimated hazard ratios are 95% confidence intervals, derived using Cox regression. Estimated hazard ratios for weeks 1-4 include the day of COVID-19 diagnosis (day 0). numerical values of estimated hazard ratios and 95% confidence intervals are displayed in Table S7.

Figure S4: Maximally adjusted hazard ratios and 95% CIs comparing the incidence of venous thrombotic events after versus before or without a COVID-19 diagnosis, in the pre-vaccination, vaccinated and unvaccinated cohorts, by subgroup.

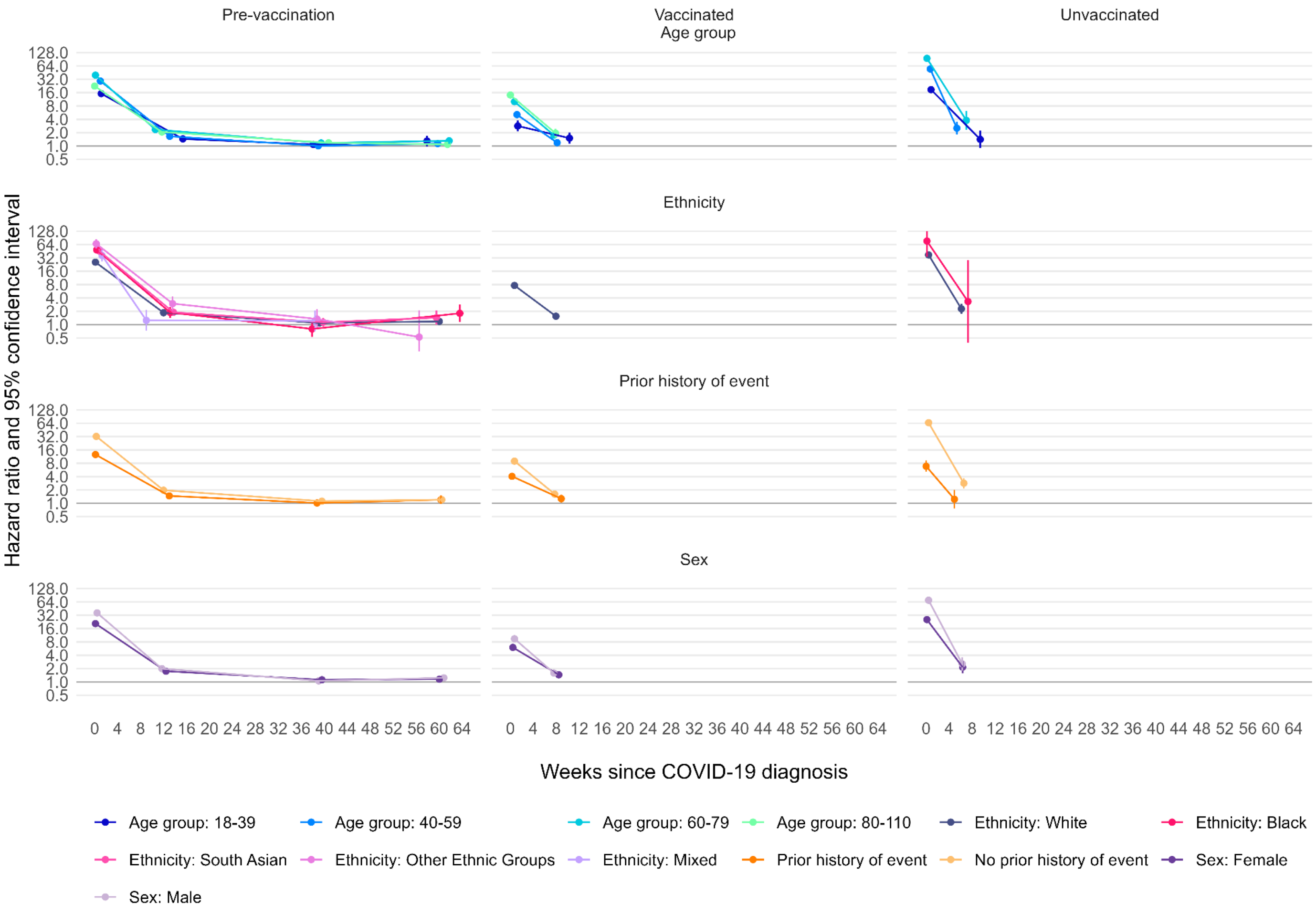

Figure S4 legend: Upper panels: Age group. Second row panels: Ethnicity. Third row panels: prior history of events. Lower panels: Sex. Left panels: pre-vaccination: Middle panels: vaccinated. Right panels: unvaccinated.

The numbers of people in the pre-vaccination, vaccinated and unvaccinated cohorts were 18,210,937; 13,572,399 and 3,161,485 respectively. The numbers of COVID-19 diagnoses were 1,150,299 in the pre-vaccination cohort, 844,235 in the vaccinated cohort and 162,103 in the unvaccinated cohort. Estimated hazard ratios are plotted at the median time of the outcome event within each follow up period in each cohort. Vertical lines around estimated hazard ratios are 95% confidence intervals, derived using Cox regression. Estimated hazard ratios for weeks 1-4 include the day of COVID-19 diagnosis (day 0). numerical values of estimated hazard ratios and 95% confidence intervals are displayed in Table S8.
