# Supplementary material for: Impact of vaccination on the association of COVID-19 with cardiovascular diseases: An OpenSAFELY cohort study
Source: Nat Commun. 2024 Mar 11;15:2173. doi: 10.1038/s41467-024-46497-0 (PMC10928172; doi:10.1038/s41467-024-46497-0)
Supplement: Supplementary file 3 — Reporting Summary [file 41467_2024_46497_MOESM3_ESM.pdf]

nature portfolio

Corresponding author(s): Professor Jonathan Sterne

Last updated by author(s): Feb 14, 2024

Reporting Summary

Nature Portfolio wishes to improve the reproducibility of the work that we publish. This form provides structure for consistency and transparency in reporting. For further information on Nature Portfolio policies, see our Editorial Policies and the Editorial Policy Checklist.

Statistics

For all statistical analyses, confirm that the following items are present in the figure legend, table legend, main text, or Methods section.

n/a	Confirmed	
☐	☒	The exact sample size (*n*) for each experimental group/condition, given as a discrete number and unit of measurement
☐	☒	A statement on whether measurements were taken from distinct samples or whether the same sample was measured repeatedly
☒	☐	The statistical test(s) used AND whether they are one- or two-sided<br>*Only common tests should be described solely by name; describe more complex techniques in the Methods section.*
☐	☒	A description of all covariates tested
☐	☒	A description of any assumptions or corrections, such as tests of normality and adjustment for multiple comparisons
☐	☒	A full description of the statistical parameters including central tendency (e.g. means) or other basic estimates (e.g. regression coefficient) AND variation (e.g. standard deviation) or associated estimates of uncertainty (e.g. confidence intervals)
☒	☐	For null hypothesis testing, the test statistic (e.g. *F*, *t*, *r*) with confidence intervals, effect sizes, degrees of freedom and *P* value noted<br>*Give P values as exact values whenever suitable.*
☒	☐	For Bayesian analysis, information on the choice of priors and Markov chain Monte Carlo settings
☐	☒	For hierarchical and complex designs, identification of the appropriate level for tests and full reporting of outcomes
☒	☐	Estimates of effect sizes (e.g. Cohen's *d*, Pearson's *r*), indicating how they were calculated

Our web collection on statistics for biologists contains articles on many of the points above.

Software and code

Policy information about availability of computer code

Data collection	Our manuscript includes the following code availability statement:<br>"All code and code lists are shared openly for review and re-use under an MIT open license (pre-vaccination cohort: https://github.com/opensafely/post-covid-Pre-vaccination-cardiovascular; vaccinated and unvaccinated cohorts: https://github.com/opensafely/post-covid-vaccinated)."<br>Our data extraction was done using Python. The corresponding code is available in the Github Repositories listed in the Code availability statement.
Data analysis	Our data curation and analysis was done in Python, R and Stata. The corresponding code is available in the Github Repositories listed in the Code availability statement i.e. https://github.com/opensafely/post-covid-Pre-vaccination-cardiovascular; https://github.com/opensafely/post-covid-vaccinated.

For manuscripts utilizing custom algorithms or software that are central to the research but not yet described in published literature, software must be made available to editors and reviewers. We strongly encourage code deposition in a community repository (e.g. GitHub). See the Nature Portfolio guidelines for submitting code & software for further information.

Data

Policy information about availability of data

All manuscripts must include a data availability statement. This statement should provide the following information, where applicable:

- Accession codes, unique identifiers, or web links for publicly available datasets
- A description of any restrictions on data availability
- For clinical datasets or third party data, please ensure that the statement adheres to our policy

The manuscript includes the following data availability statements:
"All data were linked, stored and analysed securely within the OpenSAFELY platform: https://opensafely.org/. Data include pseudonymised data such as coded diagnoses, medications and physiological parameters. No free text data are included. Detailed pseudonymised patient data is potentially re-identifiable and therefore not shared. Data can be analysed through the OpenSAFELY platform subject to appropriate agreement, approvals and training as detailed in supplemental methods."

Research involving human participants, their data, or biological material

Policy information about studies with human participants or human data. See also policy information about sex, gender (identity/presentation), and sexual orientation and race, ethnicity and racism.

Reporting on sex and gender	Sex is recorded in electronic health records.<br>Our analyses were adjusted for sex. We also present subgroup analyses by sex in supplementary material.
Reporting on race, ethnicity, or other socially relevant groupings	Ethnicity is recorded in electronic health records.<br>In our maximally adjusted models, our analysis account for ethnicity. We also present subgroup analyses by ethncity in supplementary material.
Population characteristics	age; sex; ethnicity; region; area socioeconomic deprivation; smoking status; number of GP-patient interactions in the last 12 months; and previous history of a specific comorbidity (binary) for a range of diseases (details in Table S10)
Recruitment	Cohorts are selected from linked electronic health records.
Ethics oversight	This study was approved by the Health Research Authority [REC reference 22/PR/0095] and by the University of Bristol's Faculty of Health Sciences Ethics Committee [reference 117269].

Note that full information on the approval of the study protocol must also be provided in the manuscript.

Field-specific reporting

Please select the one below that is the best fit for your research. If you are not sure, read the appropriate sections before making your selection.

☒ Life sciences ☐ Behavioural & social sciences ☐ Ecological, evolutionary & environmental sciences

For a reference copy of the document with all sections, see nature.com/documents/nr-reporting-summary-flat.pdf

Life sciences study design

All studies must disclose on these points even when the disclosure is negative.

Sample size	No sample size calculation was done as the full adult population registered with a GP practice using the TPP system was included i.e. 40% of England population. This resulted in 18,210,937 people in the pre-vaccination cohort, 13,572,399 in the vaccinated cohort, and 3,161,485 in the unvaccinated cohort.
Data exclusions	Inclusion criteria: registered with GP in the last 6 month, alive and aged between 18 and 110 years at baseline, known sex, region, area deprivation.<br>Exclusion criteria: history of Covid-19 before baseline, history of the specific cardiovascular outcome analysed except in one subgroup analysis on outcome history.<br>Additional exclusion for the vaccination cohort: a Covid-19 vaccination before Dec 8th 2020, a second dose within 3 weeks of a first dose, mixed vaccine types before May 7th 2021.<br>Additional exclusion for the unvaccinated cohort: individuals without vaccination priority group as defined by JCVI.
Replication	Data is available through the OpenSAFELY plateform https://opensafely.org/ with code and codelists available in the GitHub repositories provided above.
Randomization	NA. This was not a randomized control trial but a cohort study based on all individual level data available. Analyses were adjusted for a range of covariates as listed in "population characteristics".
Blinding	NA. This was not a randomized control trial.

Reporting for specific materials, systems and methods

We require information from authors about some types of materials, experimental systems and methods used in many studies. Here, indicate whether each material, system or method listed is relevant to your study. If you are not sure if a list item applies to your research, read the appropriate section before selecting a response.

Materials & experimental systems

n/a	Involved in the study	
☒	☐	Antibodies
☒	☐	Eukaryotic cell lines
☒	☐	Palaeontology and archaeology
☒	☐	Animals and other organisms
☐	☒	Clinical data
☒	☐	Dual use research of concern
☒	☐	Plants

Methods

n/a	Involved in the study	
☒	☐	ChIP-seq
☒	☐	Flow cytometry
☒	☐	MRI-based neuroimaging

Clinical data

Policy information about clinical studies

All manuscripts should comply with the ICMJE guidelines for publication of clinical research and a completed CONSORT checklist must be included with all submissions.

Clinical trial registration	NA.
Study protocol	https://github.com/opensafely/post-covid-vaccinated/tree/main/protocol
Data collection	Data is available through the OpenSAFELY plateform https://opensafely.org/.
Outcomes	Incidence of arterial, venous and other cardiovascular outcomes

Plants

Seed stocks	*Report on the source of all seed stocks or other plant material used. If applicable, state the seed stock centre and catalogue number. If plant specimens were collected from the field, describe the collection location, date and sampling procedures.*
Novel plant genotypes	*Describe the methods by which all novel plant genotypes were produced. This includes those generated by transgenic approaches, gene editing, chemical/radiation-based mutagenesis and hybridization. For transgenic lines, describe the transformation method, the number of independent lines analyzed and the generation upon which experiments were performed. For gene-edited lines, describe the editor used, the endogenous sequence targeted for editing, the targeting guide RNA sequence (if applicable) and how the editor was applied.*
Authentication	*Describe any authentication procedures for each seed stock used or novel genotype generated. Describe any experiments used to assess the effect of a mutation and, where applicable, how potential secondary effects (e.g. second site T-DNA insertions, mosiacism, off-target gene editing) were examined.*
